# A case of asymptomatic gastric plexiform fibromyxoma followed up for 3 years

**DOI:** 10.1002/deo2.291

**Published:** 2023-09-19

**Authors:** Naomi Sugimura, Eiji Kubota, Makiko Sasaki, Shigeki Fukusada, Yusuke Mizuno, Hiroyasu Iwasaki, Mamoru Tanaka, Keiji Ozeki, Takaya Shimura, Hiromi Kataoka

**Affiliations:** ^1^ Department of Gastroenterology and Metabolism Nagoya City University Graduate School of Medical Sciences Aichi Japan

**Keywords:** EUS‐FNA, gastric subepithelial tumor, mesenchymal tumor, plexiform fibromyxoma, submucosal dissection

## Abstract

Plexiform fibromyxoma is a rare mesenchymal tumor identified in recent years and presents as a gastrointestinal submucosal tumor that is typically located in the gastric antrum. We report a case of gastric plexiform fibromyxoma in which the diagnosis was difficult despite repeated tissue sampling. Before visiting our hospital, the patient had been followed up for 3 years without a definitive diagnosis despite serial examinations, including computed tomography, endoscopy, endoscopic ultrasound, and endoscopic ultrasound‐guided fine‐needle aspiration. Endoscopic ultrasound‐guided fine‐needle aspiration was reperformed, and endoscopic submucosal dissection for deep biopsy was conducted for differential diagnosis of the tumor. However, histological analysis with immunostaining of tumor samples obtained using these techniques cannot provide a reliable diagnosis. Finally, the tumor was resected surgically because of its increasing size, and subsequent microscopic analysis revealed a multinodular plexiform growth pattern of spindle‐like cells with myxoid stroma. Immunohistochemically, the tumor cells were positive for smooth muscle actin but negative for c‐kit, CD34, and S100. Based on these findings, the patient was diagnosed with plexiform fibromyxoma. No evidence of residual or recurrent tumors was observed at 24 months postoperatively.

## INTRODUCTION

Plexiform fibromyxoma (PF) is a rare mesenchymal tumor that was recently recognized and added to the *Classification of Tumors of the Digestive System* by the World Health Organization as a diagnostic term in 2010. PF is typically located in the gastric antrum and shows unique pathological findings, enabling differentiation from other gastric subepithelial tumors (SETs), including gastrointestinal stromal tumors.^1^ Moreover, PF is benign but is usually treated surgically because of abdominal symptoms. PF is also diagnosed histologically, supported by immunohistochemistry; thus, a sufficient tumor sample is central to diagnosis. Endoscopic biopsy, including endoscopic ultrasound (EUS)‐guided fine‐needle aspiration (EUS‐FNA), has been performed, as presented in some previous reports,^1^ but diagnosis of PF before surgical excision has been achieved in only a few cases. We report a case of PF that was followed up for 3 years without the establishment of a definitive diagnosis despite repeated EUS‐FNA and endoscopic biopsy after mucosal incision.

## CASE PRESENTATION

**FIGURE 1:**
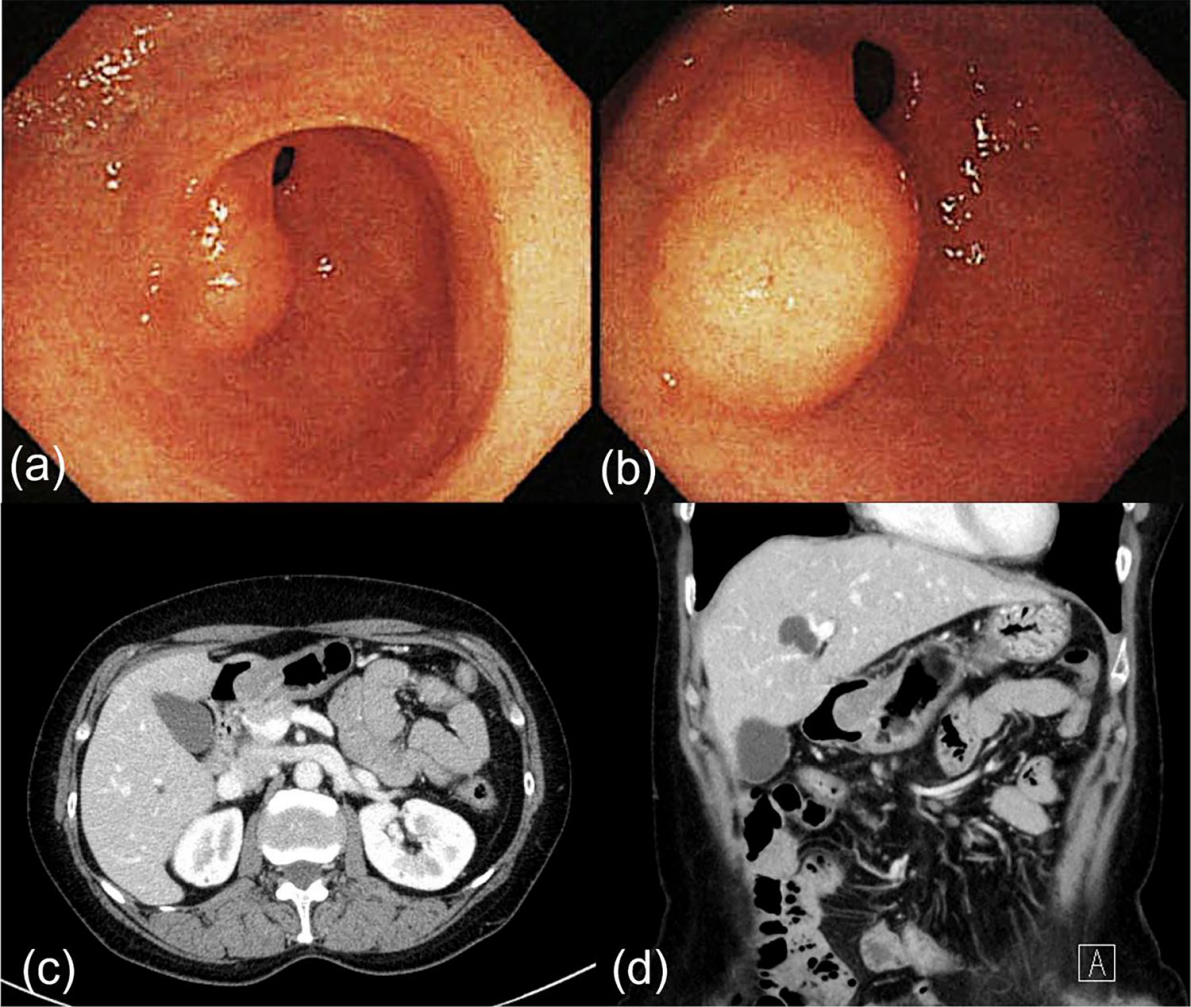
(a, b) Upper gastrointestinal endoscopy reveals a gastric submucosal tumor with a smooth surface at the gastric antrum. (c, d) Contrast‐enhanced computed tomography demonstrates a rim‐enhanced mass (20 mm in diameter) protruding into the lumen of the gastric antrum.

A 58‐year‐old Japanese woman was referred to a local core hospital because of gastric SET without symptoms. Upper gastrointestinal (GI) endoscopy revealed a tumor with a smooth surface at the gastric antrum (Figure 1a,b). Contrast‐enhanced computed tomography also revealed a 2‐cm round mass protruding into the lumen of the gastric antrum with a peripheral enhancing effect (Figure 1c,d). EUS‐FNA was performed; however, no definitive histological diagnosis was obtained. Based on these findings, he was diagnosed with benign gastric SET, which requires follow‐up by GI endoscopy and imaging modalities instead of immediate surgery.

**FIGURE 2:**
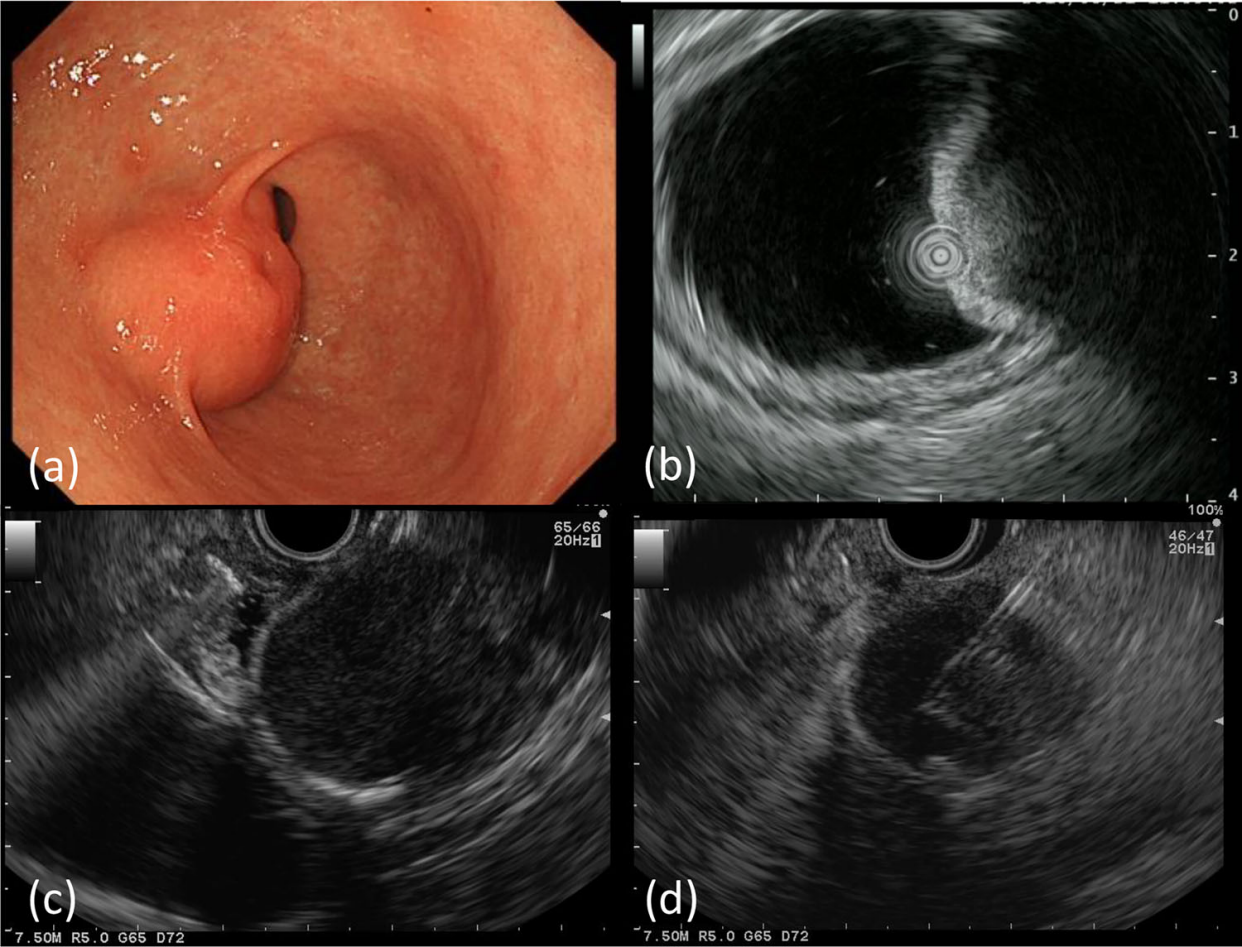
(a) Endoscopic examination shows the smooth elevated lesion with delle in the anterior wall of the gastric antrum. (b) Endoscopic ultrasound (EUS) shows a mass in the fourth layer of the gastric wall. (c) EUS shows a hypoechoic lesion that is 30 mm in diameter. (d) EUS images during EUS‐guided fine‐needle aspiration of the tumor.

**FIGURE 3:**
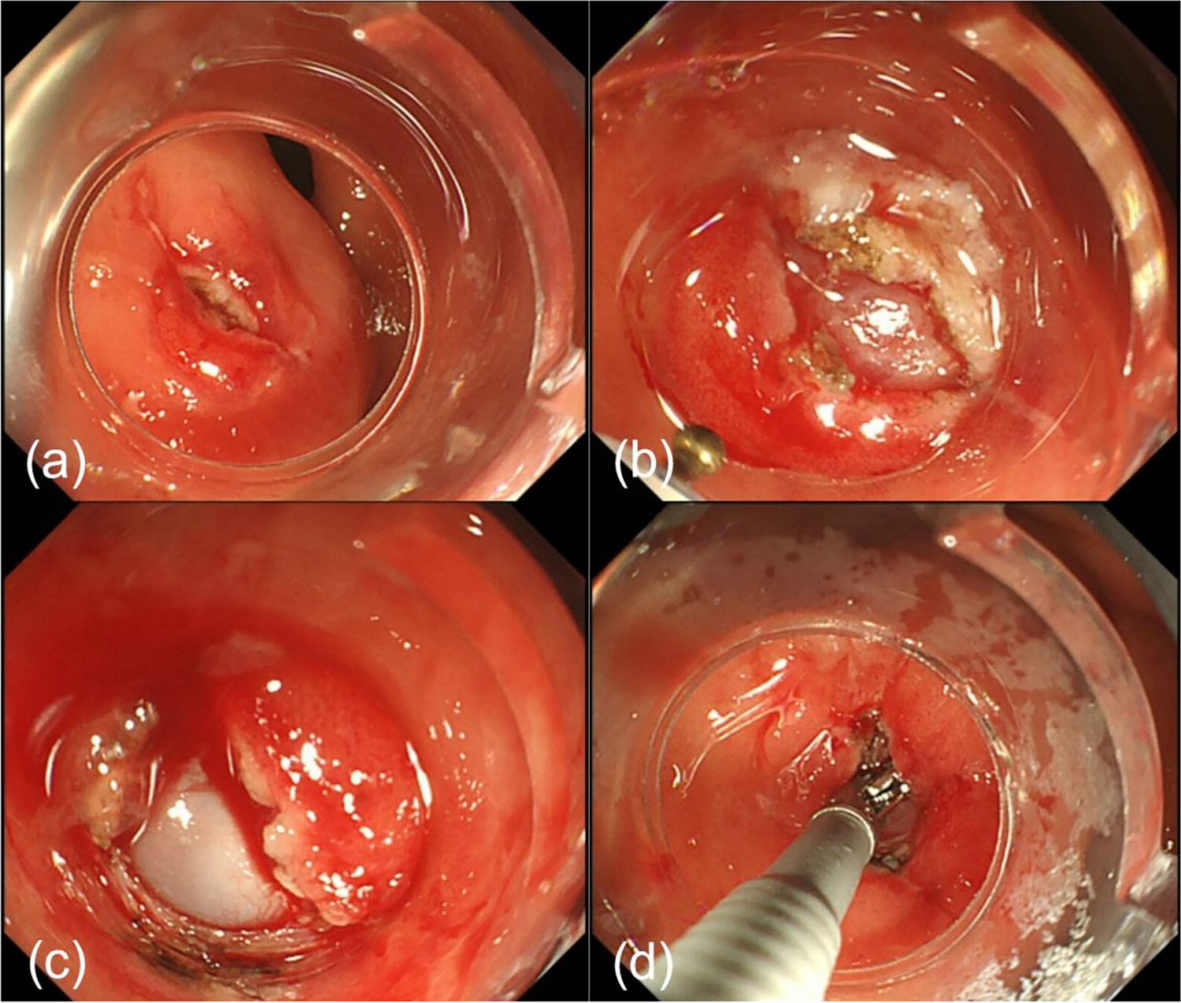
Endoscopic submucosal dissection for deep biopsy of the subepithelial tumor. (a–c) Submucosal dissection and disclosure of the subepithelial lesion using a flash knife with the aid of an ST hood. (d) Biopsy forceps are introduced through the hole, obtaining tissue samples from inside.

The patient was admitted to our hospital for surgical treatment of lung cancer 3 years after receiving a diagnosis of gastric SET. After thoracoscopic surgery for lung cancer, a follow‐up upper GI endoscopy detected an increase in the size of the SET, as a reddish‐colored tumor with a delle (Figure 2a). General examination revealed no abnormalities and laboratory data were within normal limits. EUS showed a hypoechoic mass (3 cm in diameter) in the third layer of the gastric wall (Figure 2b,c). Although EUS‐FNA was performed again (Figure 2d), the tumor sample was insufficient for diagnosis. Therefore, we also performed mucosal incision‐assisted biopsy (MIAB) for deep biopsy of the SET to obtain sufficient tumor tissue for diagnosis (Figure 3).

**FIGURE 4:**
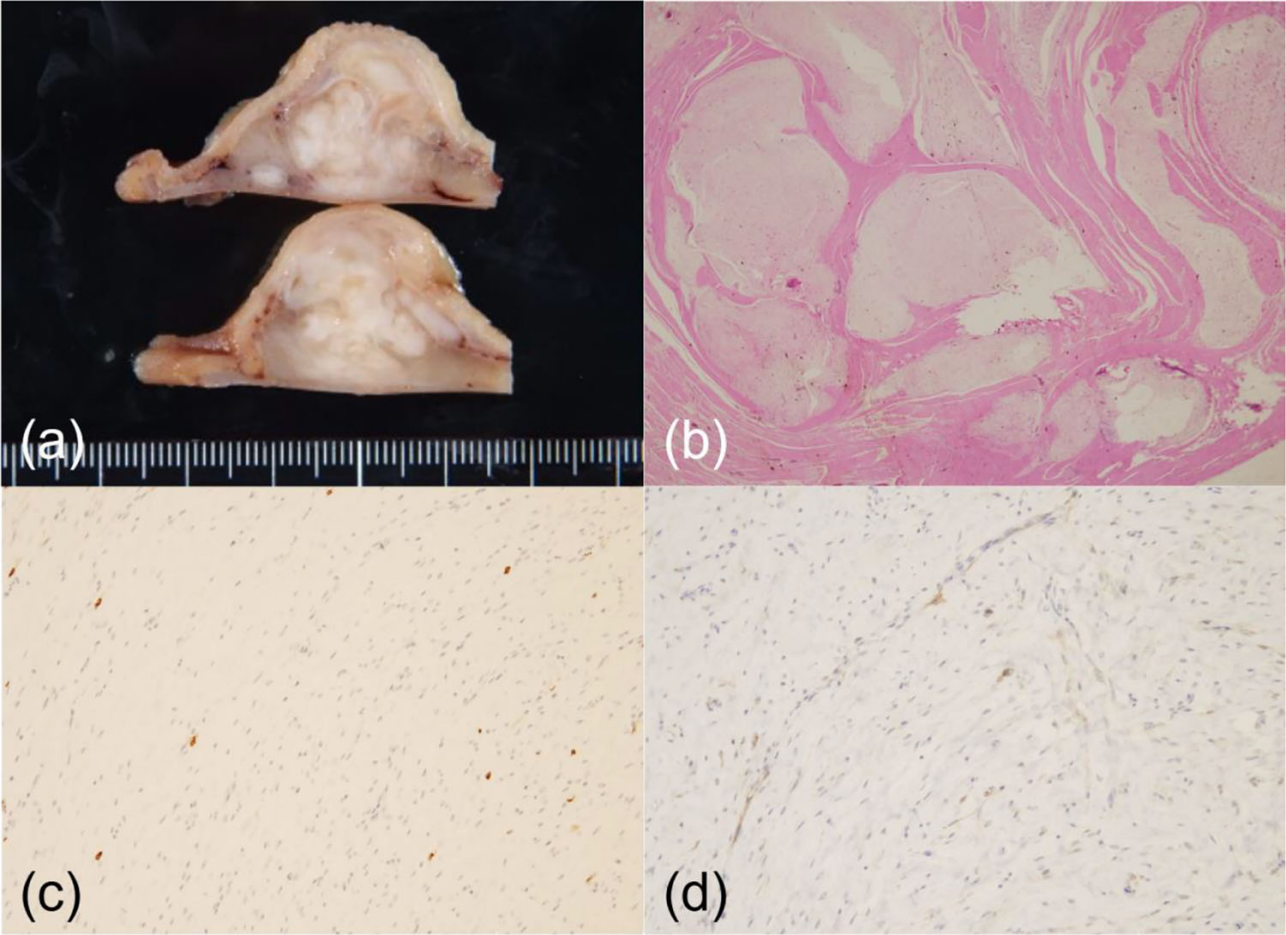
(a) The resected specimen shows a white multinodular lesion protruding into the gastric tract. (b) Hematoxylin‐eosin staining demonstrates plexiform fibromyxoma comprised of multiple myxoid nodules surrounded by the muscularis propria. (c, d) Immunostaining reveals the tumor tissue as positive for smooth muscle actin and diffusely positive for CD10.

Histological examination demonstrated a tissue sample comprised of connective tissue and fiber bundles, and immunohistochemical staining showed negative results for c‐kit, CD34, and S100, but positive results for smooth muscle actin (SMA). Although pathological findings could not provide a reliable diagnosis, the tumor's changes in size and appearance implied a malignant potential. Therefore, we decided that surgical resection was reasonable for the treatment of the patient, and distal gastrectomy of the Billroth I type was performed. The resected specimen showed a 30 × 20 × 20‐mm tumor originating from the gastric muscle layer protruding into the gastric lumen (Figure 4a). Histopathologically, spindle‐shaped cells showed a multinodular plexiform growth pattern accompanied by abundant fibrous to myxoid stroma (Figure 4b). Immunohistochemistry also revealed that the tumor cells were negative for c‐kit (not shown), diffusely positive for SMA (Figure 4c), and focally positive for CD10 (Figure 4d). CD34, MUC4, epithelial membrane antigen, and S100 were all negative (not shown). In this case, the MIB‐1 labeling index was approximately 2%. Based on these results, PF was diagnosed. The patient was discharged 2 weeks postoperatively, without any complications. No sign of disease recurrence was observed 24 months postoperatively.

## DISCUSSION

PF, also known as plexiform angiomyxoid myofibroblastic tumors, was first characterized in 2007.^2^ PF is often present in patients between 40–60 years of age and predominantly female.^1^ In this case, a 58‐year‐old woman, was considered a typical case of PF. The most common symptom of PF is abdominal pain,^1^ but our patient did not show any symptoms. PF primarily occurs in the gastric antrum (73.2%), mostly with an ulcerated surface (70.1%) and a diameter of 2–6 cm (61.2%).^1^ This case presented an approximately 3.0 cm in diameter in the gastric antrum with a nonulcerative appearance. PF usually originates from the submucosal or muscularis propria layers and appears as mildly heterogeneous hypoechoic lesions on EUS.^1^ In this case, EUS showed a hypoechoic mass located in the submucosal layer, consistent with previous reports. PF has long been considered a benign tumor; however, the first case of fatal PF recurrence and distant metastasis one year after ESD has recently been reported.^3^ Individual reports of PF demonstrating vascular and visceral invasion have also been reported,^1^ necessitating further research to determine the benign or malignant nature of PFs. Surgical excision is strongly recommended if the PF is greater than 2 cm in diameter, has high‐risk features, and tends to increase in size.^1^ Therefore, in our case, surgical treatment was rational even though a definitive diagnosis was not achieved.

The diagnosis of PF is based on histological findings supported by immunohistochemical studies. In brief, PF histologically demonstrates a multinodular, plexiform, intramural growth pattern of bland spindle cells with myxoid background.^2^ Immunohistochemically, PF shows positive results for SMA, vimentin, muscle‐specific actin, caldesmon, desmin, and CD10 but negative results for c‐kit, CD34, desmin, DOG‐1, S‐100, cytokeratin, neurofilament, β‐catenin, epithelial membrane antigen, activin receptor‐like kinase 1, CDK4, and MUC4.^4^ MIB‐1 immunoexpression, which reflects the proliferative capacity of tumor cells and is correlated with the clinical course of the tumor, is generally very low in PF.^5^ As mentioned above, evaluation of histological features and panels of immunohistochemical stains using tissue samples is essential in distinguishing PF from other gastric SETs. Accordingly, the acquisition of a sufficient tissue sample is indispensable for the accurate diagnosis of PF. In two cases with a successful preoperative diagnosis of PF by conventional endoscopic biopsy, the tumor presented as an ulcerated SET, enabling the examiners to obtain sufficient tissue samples for pathological diagnosis.^6^, ^7^ EUS‐FNA with immunohistochemical analysis was performed in the previous case, but the tumor tissues obtained by EUS‐FNA did not allow a definitive diagnosis of PF.^8^ In the present case, we could not diagnose PF despite repeated EUS‐FNA. These lines of evidence imply that EUS‐FNA cannot provide sufficient tissue for evaluating the histological features of PF. We also performed MIAB to acquire sufficient samples for a definitive diagnosis of the tumor. MIAB is reported to be superior to EUS‐FNA in the provision of sufficient material for accurate diagnosis.^9^ We identified spindle‐like cells with negative results for c‐kit and positive results for SMA in tissue samples obtained by this technique and were able to exclude the possibility that the tumor was a gastrointestinal stromal tumor. However, tumor samples were insufficient to identify the plexiform growth pattern of cells, which is a crucial histological structure of PF. Thus, endoscopic MIAB provided sufficient tissue for immunohistochemical studies, but it could not provide enough samples for microscopic detection of the plexiform growth pattern of tumor cells in our case.

Because of the limited information on PF, its pathophysiology and associated molecular alterations are not well known. Spans et al. identified overexpression of glioma‐associated oncogene homolog 1 (*GLI1*) through a recurrent metastasis‐associated lung adenocarcinoma transcript 1‐GLI1 translocation or *GLI1* upregulation in a subgroup of PFs.^10^ Molecular studies on EUS‐FNA materials seem likely to prove helpful in the diagnosis of a subset of patients with PF.

We reported a case of PF that could not be diagnosed despite serial imaging modalities and repeated tissue sampling. Previous case reports have indicated the difficulty of preoperatively diagnosing PF, even with EUS‐FNA and MIAB, which has recently been proposed as an effective and safe technique for diagnosing gastric SET.^9^ Improved preoperative diagnostic accuracy for PF requires the accumulation of cases and the development of endoscopic tissue sampling methods.

## CONFLICT OF INTEREST STATEMENT

None.
